# How should preoperative examinations be chosen for infants with a ventricular septal defect: transthoracic echocardiography, cardiac CTA, or a combination of these two technologies?

**DOI:** 10.1186/s12872-023-03635-8

**Published:** 2023-12-08

**Authors:** Wei-Hua Lin, Fu-Rong Luo, Yi-Yong Cai, Hui-Jun Xiao, Qing-Wen Huang

**Affiliations:** Department of Radiology, Zhangzhou Affiliated Hospital of Fujian Medical University, Zhangzhou, China

**Keywords:** CTA, Diagnosis, VSD, Infants

## Abstract

**Objective:**

To evaluate the accuracy of transthoracic echocardiography (TTE) and cardiac computed tomography angiography (CTA) in detecting the size and location of ventricular septal defects (VSD) in infants.

**Methods:**

Data from 258 infants diagnosed with VSD between January 2020 and December 2022 were retrospectively analyzed. All infants underwent both TTE and cardiac CTA. The accuracy of these imaging modalities was assessed by comparing their findings with intraoperative observations of VSD size and location.

**Results:**

Intraoperatively, the average VSD size was 6.1 ± 2.5 mm. The defects were classified as committed VSD (Type 1) in 45 patients, noncommitted VSD (Type 2) in 198 patients, inlet VSD (Type 3) in 12 patients, and muscular VSD (Type 4) in 3 patients. Echocardiography estimated the average VSD size at 5.6 ± 2.7 mm, with 42 patients identified as Type 1, 203 as Type 2, 10 as Type 3, and 3 as Type 4. Cardiac CTA estimated the average size at 5.9 ± 3.2 mm, with 48 patients identified as Type 1, 196 as Type 2, 11 as Type 3, and 3 as Type 4. The accuracy rates of TTE and cardiac CTA in diagnosing VSD location were 98.1% and 98.8%, respectively. A survey of surgeons indicated that 80% believe both TTE and cardiac CTA are essential preoperative evaluations.

**Conclusions:**

TTE accurately diagnoses the size and location of VSD, while cardiac CTA serves as a valuable complementary method to TTE. Most surgeons advocate for the combined use of these examinations for preoperative assessment.

## Introduction

Ventricular septal defect (VSD) is the most prevalent congenital heart disease, representing about 20–30% of all congenital heart diseases [1–4]. A detailed understanding of the location, size, and shunt direction of VSD is crucial for hemodynamic evaluation, disease assessment, and the formulation of surgical plans [5]. Transthoracic echocardiography (TTE) is extensively employed in assessing patients with congenital heart diseases due to its ease of use, portability, and lack of radiation exposure. It effectively delineates the location, size, and shunt direction of VSD [6, 7]. However, traditional TTE, being primarily two-dimensional, cannot fully depict the three-dimensional structure of the VSD [8]. Furthermore, the accuracy of echocardiography significantly depends on the echocardiographer’s experience, presenting certain limitations of TTE in clinical practice. Historically, cardiac computed tomography angiography (CTA) has been primarily used for diagnosing large vessel lesions due to concerns about radiation exposure and its limited resolution and heart rate compatibility [9]. However, recent advancements in medical imaging technology, including the use of electrocardiograph (ECG)-triggered imaging, have significantly improved the temporal resolution of cardiac CTA. These advancements have not only reduced radiation doses but also enhanced the accuracy of cardiac CTA in diagnosing intracardiac malformations. Consequently, cardiac CTA is increasingly utilized for prospective assessments of large blood vessels and airways in clinical settings [10–13]. In this study, we retrospectively analyzed the results of preoperative TTE and cardiac CTA in infants undergoing surgical repair for VSD. By comparing these results with intraoperative findings, we aim to evaluate the accuracy of these two diagnostic technologies and provide a reference for the optimal selection of preoperative examinations in infants with VSD.

## Methods and materials

Data from 258 outpatient infants diagnosed with ventricular septal defects (VSD) between January 2020 and December 2022 were retrospectively analyzed. All infants had undergone both TTE and cardiac CTA prior to surgery. The size and location of VSD confirmed intraoperatively were used as the gold standard. Additionally, a questionnaire was conducted among surgeons to determine their preferred preoperative VSD detection method in infants: transthoracic echocardiography, cardiac CTA, or a combination of the two. The inclusion criteria were as follows: infants who diagnosed with ventricular septal defects (VSD) and received further surgical repair. Exclusion criteria were as follows: (1) infants who did not undergo TTE and cardiac CTA at our hospital, (2) infants with incomplete data, and (3) cases where guardians refused participation in the study.

### Instruments and examination methods

Performed by a senior sonographer experienced in congenital heart disease using a Philips iE 33 color Doppler ultrasound device (probe frequency 2.0–5.0 MHz). Patients were positioned either supine or laterally for the echocardiographic examination. The ultrasound probe was strategically placed under the xiphoid process, at the apex, and across the third and fourth intercostal spaces and the suprasternal fossa of the left sternum. We scanned conventional sections to evaluate cardiac structures and function. In both the supine or lateral positions, a comprehensive scan included the subxiphoid artery section, short axis view of the apex, the four-chamber view, the five-chamber view, the left ventricular long axis view beside the sternum, and the short axis view near the base of the heart. These scans were crucial for assessing the integrity of the interventricular septum, as well as the type, location, size, number, and surrounding structures of the VSD. Color Doppler flow imaging facilitated observation of the direction of the intraventricular shunt, while continuous Doppler was employed to measure the shunt’s velocity and calculate the maximum pressure gradient.

Conducted by a senior radiologist, using a 256-row GE Revolution CT scanner for prospective ECG-gated axial scanning. The patients were scanned in a supine position, from the thoracic entrance to the diaphragmatic surface. The scanning parameters were set as follows: tube voltage at 70 Kv, tube current between 50 and 240 mA, and a ball tube rotation time of 0.28 s per turn, and the noise index was 20. For the contrast agent, an iodine-based solution (300 mgI/mL) was administered via a double-barrel high-pressure syringe. The dosage was 1.5 mL/kg, with an injection rate of 0.8–1.4 mL/s, followed by a 10 mL normal saline flush at the same rate. The descending aorta at the level of the four chambers of the heart served as the monitoring level, utilizing the Bolus Tracking technique for manual triggering. Images were reconstructed using the iterative reconstruction technique (ASIR-V) set at 70%. The reconstructed layer thickness and spacing were both 0.625 mm, with an interval set at 5% of the R-R interval. Reconstruct 30–85% of the data to identify the phase with minimal motion artifacts, and transfer it to the GE 4.7 workstation for post-processing and diagnostic assessment. The optimal time for image acquisition was determined using multiplanar reconstruction (MRP) and maximum intensity projection (MIP). Additional techniques like volume rendering (VR) were employed to reconstruct the plane that best displayed the largest diameter of the VSD from multiple directions and angles. The maximum diameter of the VSD was measured, and the location of the VSD was classified.

### VSD classification

Based on the varied morphologies of VSD, we adopted the classification system proposed by David Nau et al. The largest measurable diameter of the VSD was used to represent its size [14]. The localization of VSD was categorized into four main types according to Jacobs’ classification: (1) Committed VSD (Type 1), located beneath the semilunar valves in the conal or outlet septum; (2) Noncommitted VSD (Type 2), confluent with and involving the membranous septum, bordered by an atrioventricular valve, but not near the great vessels; (3) Inlet VSD (Type 3), situated in the inlet of the right ventricular septum, immediately inferior to the atrioventricular valve apparatus; and (4) Muscular VSD (Type 4), entirely surrounded by muscle [15].

### The questionnaire survey

A custom questionnaire was developed to explore the preferences of 10 cardiac surgeons regarding preoperative examination methods for infants with ventricular septal defects (VSD). The questionnaire included the following questions: (1) Do you believe cardiac CTA can accurately assess the size of VSD? (2) Do you believe cardiac CTA can accurately determine the location of VSD? (3) Do you think color echocardiography can accurately measure the size of VSD? (4) Do you think color echocardiography can accurately determine the location of VSD? (5) Is performing only TTE sufficient before surgery for infants with VSD? (6) Is performing only cardiac CTA sufficient before surgery for infants with VSD? (7) How do you decide between TTE, cardiac CTA, or a combination of these technologies for the preoperative examination of infants with VSD?

### Statistical analysis

Statistical analysis was performed using SPSS software (version 25.0). Quantitative data were expressed as mean ± standard deviation. Analysis of variance (ANOVA) was utilized to compare means across three groups. The S-N-K (Student-Newman-Keuls) method was employed for the comparison of qualitative data between groups. A p-value of less than 0.05 was considered statistically significant.

## Results

All patients successfully underwent both TTE and cardiac CTA prior to surgery. The cohort consisted of 132 males and 126 females with an average age of 6.2 ± 4.6 months and a body weight of 6.5 ± 3.1 kg. Among them, 25 patients had additional intracardiac malformations, including pulmonary stenosis (8 cases), mitral insufficiency (5 cases), and right ventricular outflow tract stenosis (12 cases). Additionally, 23 patients presented with extracardiac malformations, comprising coarctation of the aorta (11 cases), partial pulmonary vein drainage (1 case), tracheal stenosis (4 cases), and vascular ring combined with tracheal stenosis (5 cases).

Intraoperative observations revealed an average VSD size of 6.1 ± 2.5 mm. The distribution of VSD types was as follows: Type 1 (committed VSD) in 45 patients, Type 2 (noncommitted VSD) in 198 patients, Type 3 (inlet VSD) in 12 patients, and Type 4 (muscular VSD) in 3 patients. Echocardiographic assessment estimated the average VSD size at 5.6 ± 2.7 mm (Type 1 in 42 patients, Type 2 in 203 patients, Type 3 in 10 patients, and Type 4 in 3 patients). Cardiac CTA estimated the average size at 5.9 ± 3.2 mm (Type 1 in 48 patients, Type 2 in 196 patients, Type 3 in 11 patients, and Type 4 in 3 patients). There was no significant difference in the size and location of VSD as diagnosed by the two technologies. The diagnostic accuracy of TTE for VSD location was 98.1%, while that of cardiac CTA was 98.8%, with no significant difference in accuracy between the two methods.

The questionnaire results indicated that all surgeons agreed on the accuracy of TTE in determining the size and location of VSD. Eight surgeons opined that cardiac CTA could accurately measure the size and determine the location of VSD, while two surgeons disagreed. Regarding the selection of preoperative examinations, two surgeons preferred using only TTE, eight advocated for the combined use of TTE and cardiac CTA, and none favored the exclusive use of cardiac CTA.

## Discussions

Surgical repair remains the primary treatment modality for VSD [1–4]. Accurate assessment of VSD size and location is critical in formulating a surgical plan. TTE is the most commonly used preoperative examination for congenital heart defects, acclaimed for its ease of use, non-invasiveness, and independence from heart rate variability. Numerous studies, as well as our own findings, affirm that TTE can accurately measure the size and location of VSD, alongside dynamically assessing heart valve function, cardiac contractility, and pulmonary artery pressure. This comprehensive evaluation is particularly crucial in cases of cardiac malformations [6, 8, 16].

This study demonstrated that the size and location of VSD as measured by TTE were consistent with intraoperative findings, indicating high accuracy in diagnosing VSD and other intracardiac malformations. However, TTE has its limitations: (1) It produces two-dimensional images, necessitating the synthesis of multiple sections for a three-dimensional conceptualization of VSD. (2) Its diagnostic accuracy is influenced by the sternum and lungs and depends greatly on the cardiac sonographer’s skill and experience, affecting its reproducibility and utility. (3) TTE’s ability to assess extra-cardiac vascular diseases is limited, which is significant considering the prevalence of extra-cardiac vascular complications in infants with VSD. (4) TTE cannot evaluate tracheal conditions. For instance, in this study, three patients diagnosed with mild aortic stenosis by TTE prior to surgery for coarctation of the aorta displayed more severe conditions intraoperatively. Additionally, nine patients had tracheal stenosis, a condition undetectable by TTE.

In recent years, cardiac CTA has experienced significant technological advancements, enabling detailed cardiac structural scanning and reconstruction for congenital heart disease with reduced radiation exposure [17, 18]. Its superior spatial and temporal resolution, short scan duration, prospective ECG gating, and advanced iterative reconstruction post-processing make cardiac CTA an excellent tool for the morphological assessment of congenital heart diseases. Notably, it can effectively visualize the three-dimensional morphology of VSD and its spatial relationship with surrounding tissues. This study found that cardiac CTA’s measurements of VSD size and location align with intraoperative findings, confirming its accuracy in delineating these parameters. Furthermore, cardiac CTA is considered the gold standard for diagnosing vascular malformations, offering precise representations of adjacent large vessels. It also provides valuable images of the trachea and lungs, aiding in the evaluation and detection of related diseases. However, the potential for radiation exposure remains a concern [19, 9]. Studies have indicated DNA damage and cell apoptosis at exposures exceeding 7.5 mSv [20]. To mitigate this risk, we employed low-tube voltage, prospective ECG gating, and iterative reconstruction techniques, achieving low-dose scans with radiation levels between 0.48 and 4 mSv. Despite these advances, cardiac CTA cannot dynamically assess heart valve conditions, pulmonary artery pressure, or cardiac contractility. Additionally, performing cardiac CTA in infants often requires sedation due to the need for patient cooperation during the procedure.

TTE and cardiac CTA each have distinct advantages and limitations in diagnosing congenital heart disease. In our survey focusing on the preoperative examination of infants with VSD, three surgeons opined that only TTE was necessary before surgery, deeming cardiac CTA unnecessary. Conversely, seven surgeons recommended a combination of both TTE and cardiac CTA for preoperative evaluation. None believed that cardiac CTA alone was sufficient. Although cardiac CTA provides accurate assessments of VSD size and location, it produces mainly static images and lacks the capability to dynamically monitor cardiac functions like valve activity, pulmonary artery pressure, and cardiac contractility, as TTE does. All surveyed cardiac surgeons agreed that cardiac CTA alone was inadequate for preoperative evaluation. However, two surgeons felt that preoperative CTA was not essential, stipulating that TTE must be performed by a highly skilled cardiac sonographer and should be supplemented with a lung CT scan to rule out pulmonary and tracheal issues. The majority, eight surgeons, advocated for the combined use of TTE and cardiac CTA before surgery. They believed this approach would leverage the strengths of both modalities, enabling accurate assessments of VSD’s size, location, and three-dimensional shape, identifying other cardiac and tracheal malformations, and facilitating comprehensive preoperative planning.

This study has certain limitations that must be considered. Firstly, it is a retrospective analysis with a relatively small sample size, which may affect the generalizability of the results. Secondly, the measurement of VSD sizes during surgery, performed in a state of cardiac arrest, might be influenced by nonphysiological dilation of the ventricles. This factor could introduce a bias in the assessment of VSD dimensions.

## Conclusions

TTE not only accurately diagnoses the size and location of VSD but also evaluates heart valve conditions, pulmonary artery pressure, and cardiac contractility and relaxation. Cardiac CTA serves as a valuable complementary method to TTE. It not only corroborates TTE’s findings but also provides additional information, including the detection of macrovascular malformations and airway abnormalities.

## Data Availability

The datasets used and analyzed during the current study are available from the corresponding author on reasonable request.

## Abbreviations

VSD: Ventricular septal defect
TTE: Transthoracic echocardiography
CTA: Computed tomography qngiography
ECG: Electrocardiograph

## References

[1] Anderson RH, Becker AE, Tynan M (1986). Description of ventricular septal defects–or how long is a piece of string?. Int J Cardiol.

[2] Hoffman JI, Kaplan S (2002). The incidence of congenital Heart Disease. J Am Coll Cardiol.

[3] Xing Q, Pan S, An Q, Zhang Z, Li J, Li F, Wu Q, Zhuang Z (2010). Minimally invasive periventricular device closure of perimembranous ventricular septal defect without cardiopulmonary bypass: multicenter experience and mid-term follow-up. J Thorac Cardiovasc Surg.

[4] Zhu D, Gan C, Li X, An Q, Luo S, Tang H, Feng Y, Lin K (2013). Perventricular device closure of perimembranous ventricular septal defect in pediatric patients: technical and morphological considerations. Thorac Cardiovasc Surg.

[5] Sun R, Liu M, Lu L, Zheng Y, Zhang P (2015). Congenital Heart Disease: causes, diagnosis, symptoms, and treatments. Cell Biochem Biophys.

[6] Santoro G, Marino B (1994). Reliability of echocardiography for the evaluation of VSD anatomy in tetralogy of Fallot. Int J Cardiol.

[7] Gurgun C, Ozerkan F, Akin M (2000). Ruptured Aneurysm of sinus of Valsalva with ventricular septal defect: the role of transesophageal echocardiography in diagnosis. Int J Cardiol.

[8] Chen FL, Hsiung MC, Nanda N, Hsieh KS, Chou MC (2006). Real time three-dimensional echocardiography in assessing ventricular septal defects: an echocardiographic-surgical correlative study. Echocardiography.

[9] Earls JP, Berman EL, Urban BA, Curry CA, Lane JL, Jennings RS, McCulloch CC, Hsieh J, Londt JH (2008). Prospectively gated transverse coronary CT angiography versus retrospectively gated helical technique: improved image quality and reduced radiation dose. Radiology.

[10] Goo HW, Park IS, Ko JK, Kim YH, Seo DM, Yun TJ, Park JJ, Yoon CH. CT of congenital Heart Disease: normal anatomy and typical pathologic conditions. Radiographics. 2003;23 Spec No:S147-65. 10.1148/rg.23si035501 14557509

[11] Eom HJ, Yang DH, Kang JW, Kim DH, Song JM, Kang DH, Song JK, Kim JB, Jung SH, Choo SJ, Chung CH, Lee JW, Lim TH (2015). Preoperative cardiac computed tomography for demonstration of congenital cardiac septal defect in adults. Eur Radiol.

[12] Lee T, Tsai IC, Fu YC, Jan SL, Wang CC, Chang Y, Chen MC (2006). Using multidetector-row CT in neonates with complex congenital Heart Disease to replace diagnostic cardiac catheterization for anatomical investigation: initial experiences in technical and clinical feasibility. Pediatr Radiol.

[13] Han BK, Overman DM, Grant K, Rosenthal K, Rutten-Ramos S, Cook D, Lesser JR. Non-sedated, free breathing cardiac CT for evaluation of complex congenital Heart Disease in neonates. J Cardiovasc Comput Tomogr. 2013 Nov-Dec;7(6):354–60. 10.1016/j.jcct.2013.11.006 24331930

[14] Nau D, Wuest W, Rompel O, Hammon M, Gloeckler M, Toka O, Dittrich S, Rueffer A, Cesnjevar R, Lell MM, Uder M, May MS. Evaluation of ventricular septal defects using high pitch computed tomography angiography of the chest in infants with complex congenital heart defects below one year of age. J Cardiovasc Comput Tomogr. 2019 Jul-Aug;13(4):226–33. 10.1016/j.jcct.2019.01.023 30737152

[15] Jacobs JP, Burke RP, Quintessenza JA, Mavroudis C (2000). Congenital heart Surgery nomenclature and database project: ventricular septal defect. Ann Thorac Surg.

[16] Pettersen MD, Du W, Skeens ME, Humes RA (2008). Regression equations for calculation of z scores of cardiac structures in a large cohort of healthy infants, children, and adolescents: an echocardiographic study. J Am Soc Echocardiogr.

[17] Sugeng L, Mor-Avi V, Weinert L, Niel J, Ebner C, Steringer-Mascherbauer R, Bartolles R, Baumann R, Schummers G, Lang RM, Nesser HJ. Multimodality comparison of quantitative volumetric analysis of the right ventricle. JACC Cardiovasc Imaging. 2010;3 (1): 10 to 8. 10.1016/j.jcmg.2009.09.017 20129525

[18] Yamasaki Y, Nagao M, Yamamura K, Yonezawa M, Matsuo Y, Kawanami S, Kamitani T, Higuchi K, Sakamoto I, Shiokawa Y, Yabuuchi H, Honda H (2014). Quantitative assessment of right ventricular function and pulmonary regurgitation in surgically repaired tetralogy of fallot using 256-slice CT: comparison with 3-Tesla MRI. Eur Radiol.

[19] Hunink MG, Gazelle GS (2003). CT screening: a trade-off of risks, benefits, and costs. J Clin Invest.

[20] Nguyen PK, Lee WH, Li YF, Hong WX, Hu S, Chan C, Liang G, Nguyen I, Ong SG, Churko J, Wang J, Altman RB, Fleischmann D, Wu JC (2015). Assessment of the Radiation effects of Cardiac CT Angiography using protein and genetic biomarkers. JACC Cardiovasc Imaging.

